# Supplementary material for: Modulating oxygen coverage of Ti3C2Tx MXenes to boost catalytic activity for HCOOH dehydrogenation
Source: Nat Commun. 2020 Aug 25;11:4251. doi: 10.1038/s41467-020-18091-7 (PMC7447762; doi:10.1038/s41467-020-18091-7)
Supplement: Supplementary file 1 — Supplementary Information [file 41467_2020_18091_MOESM1_ESM.pdf]

Supplementary Information for

“Modulating Oxygen Coverage of $Ti_3C_2T_x$ MXenes to Boost Catalytic Activity for HCOOH Dehydrogenation”

Tingting Hou et al.

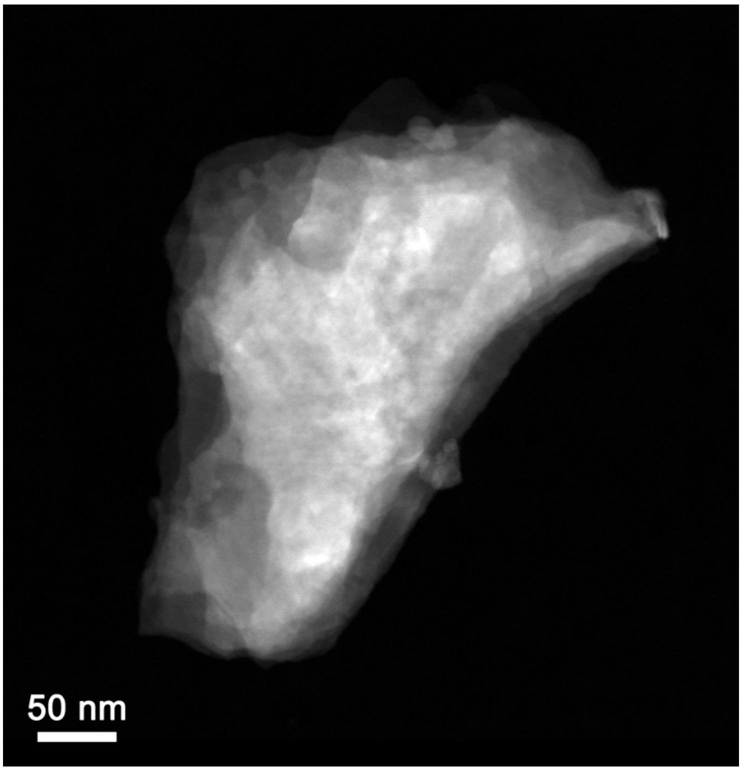

Supplementary Figure 1. HAADF-STEM image of $Ti_3C_2T_x$-25.

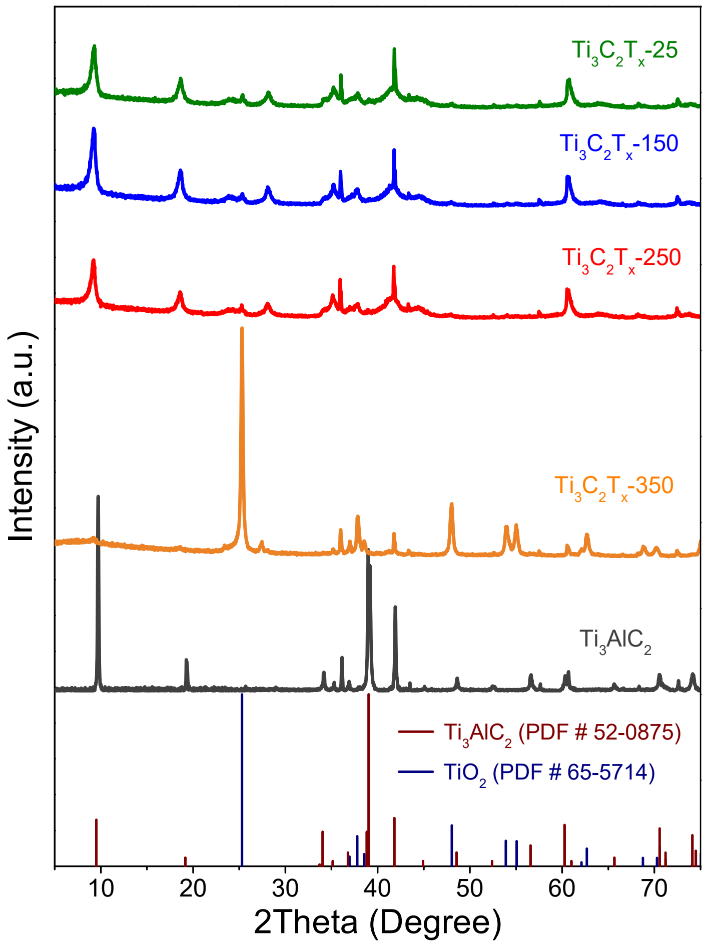

Supplementary Figure 2. XRD patterns of $Ti_3C_2T_x$-25, $Ti_3C_2T_x$-150, $Ti_3C_2T_x$-250, $Ti_3C_2T_x$-350, and Ti_3AlC_2.

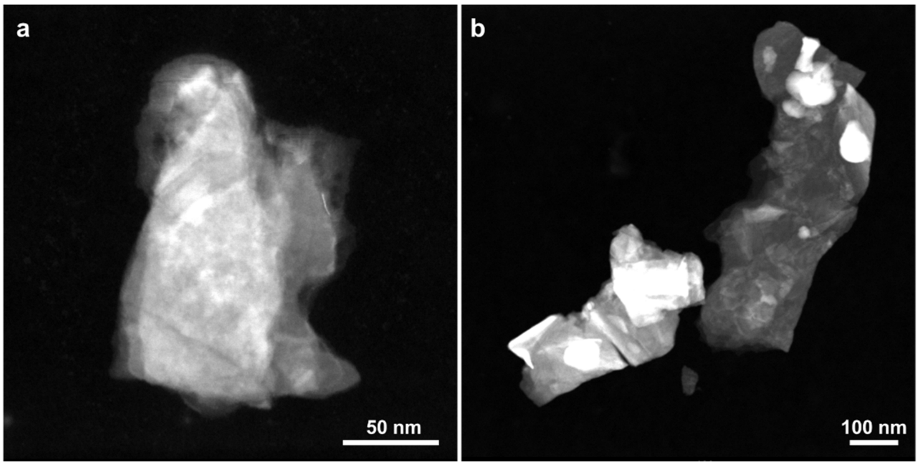

Supplementary Figure 3. HAADF-STEM images of (**a**) $Ti_3C_2T_x$-150 and (**b**) $Ti_3C_2T_x$-350.

Supplementary Table 1. The BET surface areas of different catalysts.

Catalyst	BET surface areas (m^2/g)
$Ti_3C_2T_x$-25	16
$Ti_3C_2T_x$-150	20
$Ti_3C_2T_x$-250	21
$Ti_3C_2T_x$-350	44

Supplementary Table 2. EXAFS fitting parameters at the Ti K-edge for the obtained samples (S_0^2=0.838).

Sample	Shell	*CN*	*R*(Å)	*D. W.*	ΔE_0 (eV)	*R* factor
TiO_2	Ti-O	6	2.00	0.0059	1.2	0.0063
	Ti-Ti	4	2.86	0.0086		
TiC	Ti-C	6	2.24	0.0043	-6.1	0.0081
	Ti-Ti	12	3.06	0.0034		
$Ti_3C_2T_x$-25	Ti-C/O	4.5	2.13	0.0089	-2.2	0.0006
	Ti-Ti	5.4	3.01	0.0058		
$Ti_3C_2T_x$-150	Ti-C/O	4.7	2.13	0.0080	-3.6	0.0008
	Ti-Ti	5.6	3.01	0.0053		
$Ti_3C_2T_x$-250	Ti-C/O	4.9	2.14	0.0085	-4.2	0.0008
	Ti-Ti	5.3	3.02	0.0052		
$Ti_3C_2T_x$-350	Ti-C/O	2.9	1.97	0.0007	-0.6	0.0165
	Ti-Ti	2.4	3.02	0.0050		

CN, coordination number; *R*(Å), bond distance; *D. W.,* Debye-Waller factor; ΔE_0, the inner potential correction to account for the difference in the inner potential between the sample and the reference compound; *R* factor, goodness of fit. S_0^2 was set to be 0.838, according to the experimental EXAFS fit of TiO_2 reference by fixing CN as the known crystallographic value.

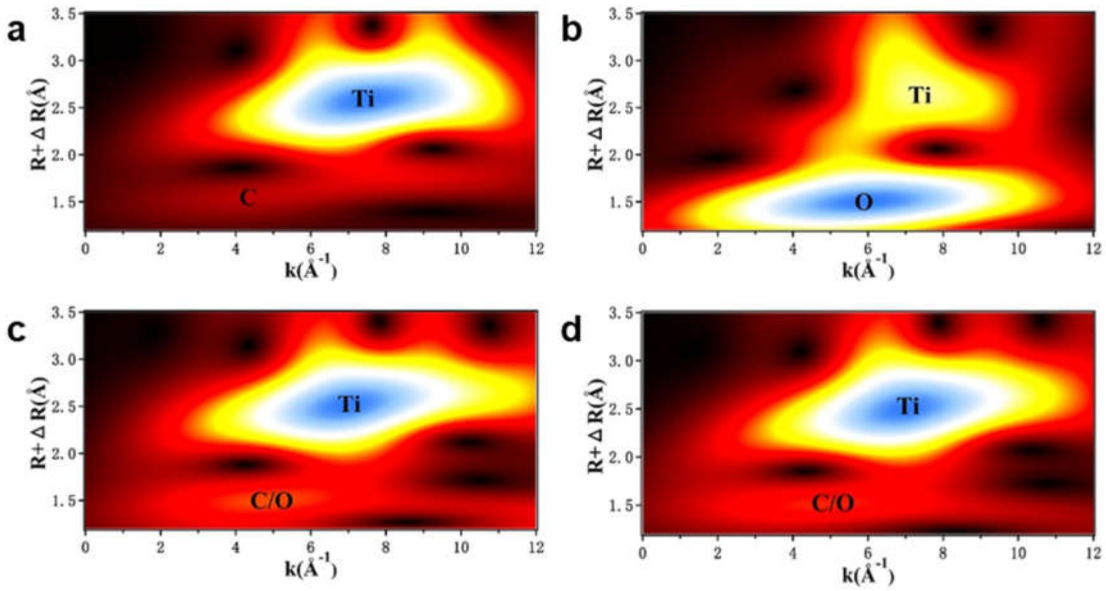

Supplementary Figure 4. The wavelet transform analysis in Ti K-edge for (**a**) TiC, (**b**) TiO_2, (**c**) $Ti_3C_2T_x$-25, and (**d**) $Ti_3C_2T_x$-150.

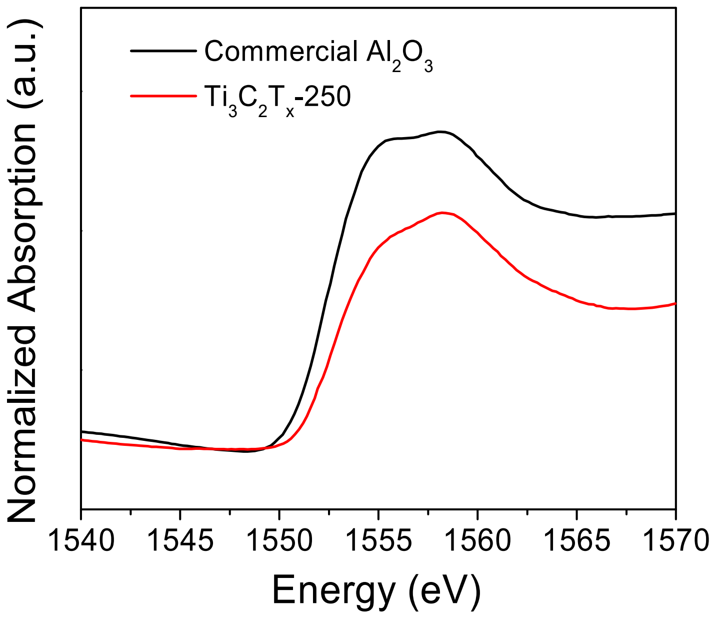

Supplementary Figure 5. Al K edge XANES spectra for $Ti_3C_2T_x$-250 (Commercial Al_2O_3 was used as reference).

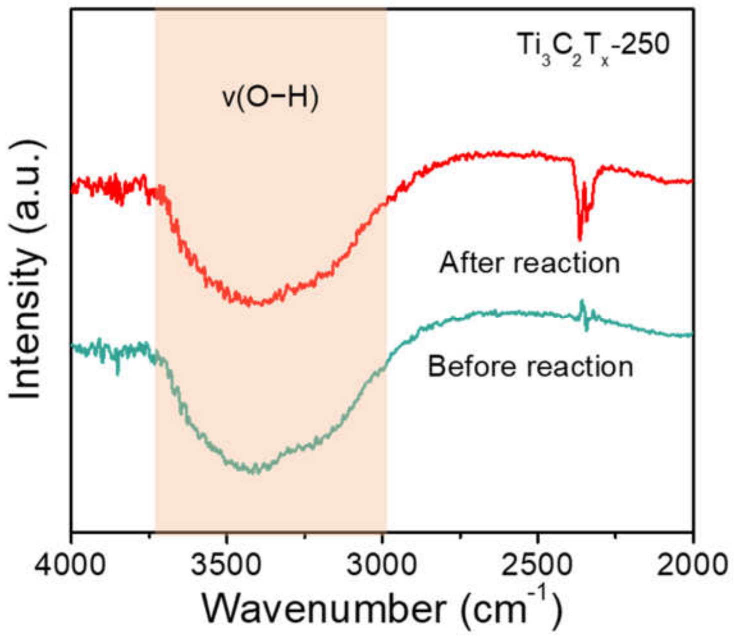

Supplementary Figure 6. DRIFT spectra of $Ti_3C_2T_x$-250 before and after catalytic test. The absorption peak ranging from 3000 to 3700 cm^{-1} was assigned to the ν(O−H) stretching mode of surface OH groups.

Supplementary Table 3. The percentages of O-Ti species for different samples determined by XPS spectra of O 1*s*.

Sample	The percentages of O-Ti species (%)
$Ti_3C_2T_x$-25	3.4
$Ti_3C_2T_x$-150	7.3
$Ti_3C_2T_x$-250	26.5

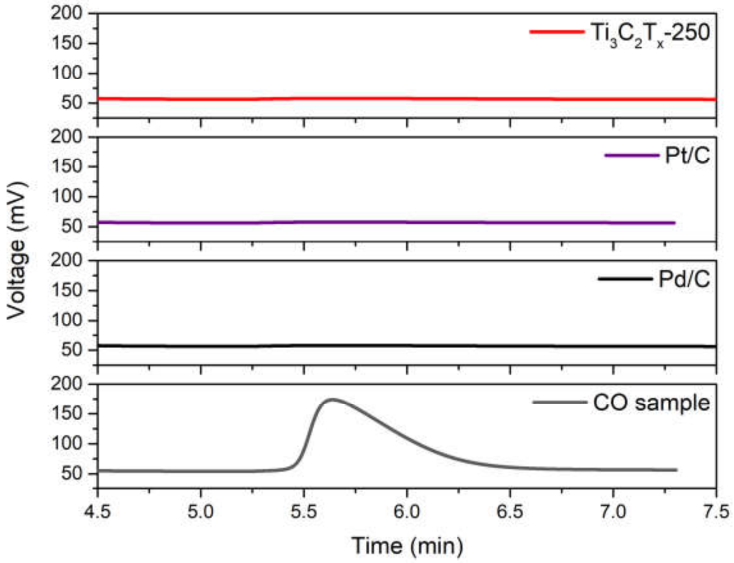

Supplementary Figure 7. The gas chromatogram profiles for the detection of CO.

Supplementary Table 4. Catalytic performance for the dehydrogenation of formic acid by representative noble-metal based catalysts.[S1-S13]

Catalyst[(a)]	Reaction temperature (°C)	Performance[(c)]	Ref.
Soybean-Mo (0.1)[(b)]	110	90%[(e)]	*Energ. Convers. Manage.* **2018**, *164*, 122
Co(1)/phen(7)/C[(b)]	110	220 h^{-1}	*Angew. Chem. Int. Edit.* **2017**, *56*, 16616
Se-doped graphene/$CoFe_2O_4$[(b)]	60	306 h^{-1}	*Int. J. Hydrogen Energ.* **2016**, *41*, 20147
Fe/*rac*-P4	60	1853 h^{-1}	*ACS Catal.* **2015**, *5*, 1254
CdS nanocrystals[(b)]	25	116 mmol H_2 g^{-1} h^{-1}[(d)]	*Angew. Chem. Int. Edit.* **2015**, *54*, 9627
Ni pincer complex	60	209 h^{-1}	*Chemcatchem* **2015**, *7*, 65
Aliphatic Fe pincer complex	80	622 h^{-1}	*J. Am. Chem. Soc.* **2014**, *136*, 10234
Al–PNP pincer complex	65	5200 h^{-1}	*Chem. Sci.* **2014**, *5*, 2771
MoS_2/Graphene[(b)]	235	92%[(e)]	*ACS Catal.* **2014**, *4*, 3950
Fe-PNP pincer complex	65	836 h^{-1}	*Chem. Eur. J.* **2013**, *19*, 8068
$[Fe(BF_4)_2]6H_2O/PP_3$	80	9425 h^{-1}	*Science* **2011**, *333*, 1733
$[Fe(CO)_3(PBn_3)_2]$	60	247[(f)]	*Angew. Chem., Int. Ed.* ***2010**, 49, 8993-8996*
$Fe_3(CO)_{12}/PPh_3$/tpy	40	200 h^{-1}	*J. Am. Chem. Soc.* ***2010**, 132, 8924–8934*
$Ti_3C_2T_x$-200[(b)]	80	365 mmol H_2 g^{-1} h^{-1}[(d)] 94%[(e)]	*This work*

[(a)]Homogeneous catalyst unless specified; [(b)]heterogeneous catalyst; [(c)]TOF unless specified; [(d)]mass activity; [(e)]conversion; [(f)]TON.

Supplementary Table 5. The calculated TOFs of $Ti_3C_2T_x$-250, Pd/C, and Pt/C in HCOOH dehydrogenation.

Samples	TOF (h^{-1})
$Ti_3C_2T_x$-250	960
Pd/C	2679
Pt/C	4585

The surface Ti species were regarded as the active sites. TOF of $Ti_3C_2T_x$-250 in HCOOH dehydrogenation was calculated to be 960 h^{-1} according to the following formula:

$$TOF = \frac{N_{HCOOH}}{N_{surface\ Ti}} = \frac{Mass\ activity \times m_{Ti_3C_2T_x-250} \times N_A}{A_{Ti_3C_2T_x-250} \times m_{Ti_3C_2T_x-250} \times \rho_{surface\ Ti}}$$

Where Mass activity, $m_{Ti3C2Tx\text{-}250}$ and N_A were 365 mmol/g/h, 30 mg, and 6.02×10^23. $A_{Ti3C2Tx\text{-}250}$ and $\rho_{surface\ Ti}$ was 21 m^2/g and 1.09091E+19 m^{-2} determined by BET surface area and crystalline structure.

As for Pd/C and Pt/C, TOFs were calculated to be 2679 and 4585 h^{-1} according to the following formula, respectively:

$$TOF = \frac{N_{HCOOH}}{N_{surface\ Pd\ or\ Pt}} = \frac{Mass\ activity \times m_{Ti_3C_2T_x-250} \times N_A}{N_{surface\ Pd\ or\ Pt}}$$

Where $N_{surface\ Pd\ or\ Pt}$ was determined by CO pulse chemisorption.

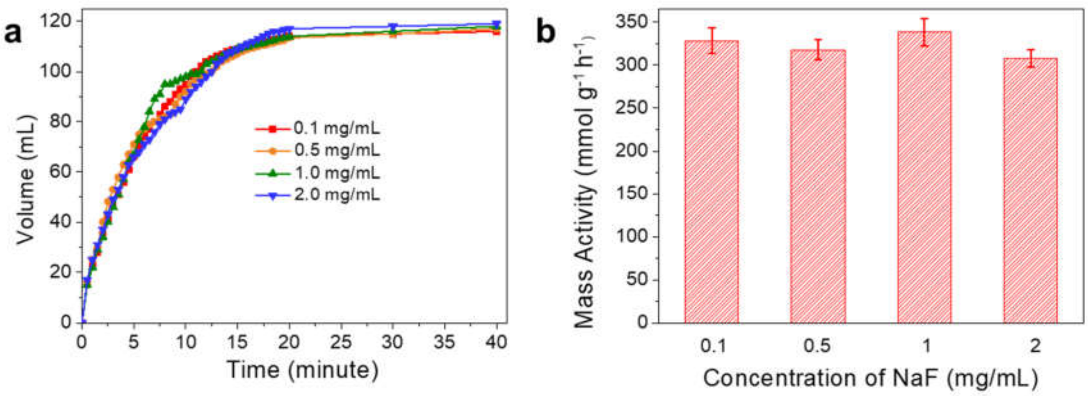

Supplementary Figure 8. Comparison of the (A) volume of the generated gas *vs* reaction time and (B) the mass activity of $Ti_3C_2T_x$-250 for HCOOH dehydrogenation in NaF aqueous solution with different concentrations.

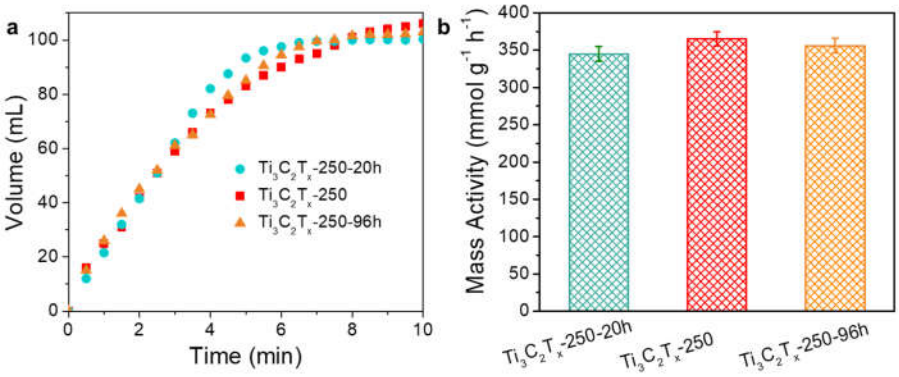

Supplementary Figure 9. Comparison of (**a**) the volume of the generated gas *vs* reaction time and (**b**) the mass activities of $Ti_3C_2T_x$-250-20h, $Ti_3C_2T_x$-250, and $Ti_3C_2T_x$-250-96h for HCOOH dehydrogenation. $Ti_3C_2T_x$-250-20h, $Ti_3C_2T_x$-250, and $Ti_3C_2T_x$-250-96h were prepared with the similar method except for the different etching time of 20, 72 and 96 h, respectively.

Supplementary Table 6. The activation energies of different catalysts for the dehydrogenation of HCOOH determined by Arrhenius plots**.**

Sample	Ea (KJ/mol)
$Ti_3C_2T_x$-25	46.4±4.9
$Ti_3C_2T_x$-150	44.6±4.4
$Ti_3C_2T_x$-250	31.4±4.9
$Ti_3C_2T_x$-350	39.7±4.9
Pt/C	39.1±2.7
Pd/C	39.8±2.4

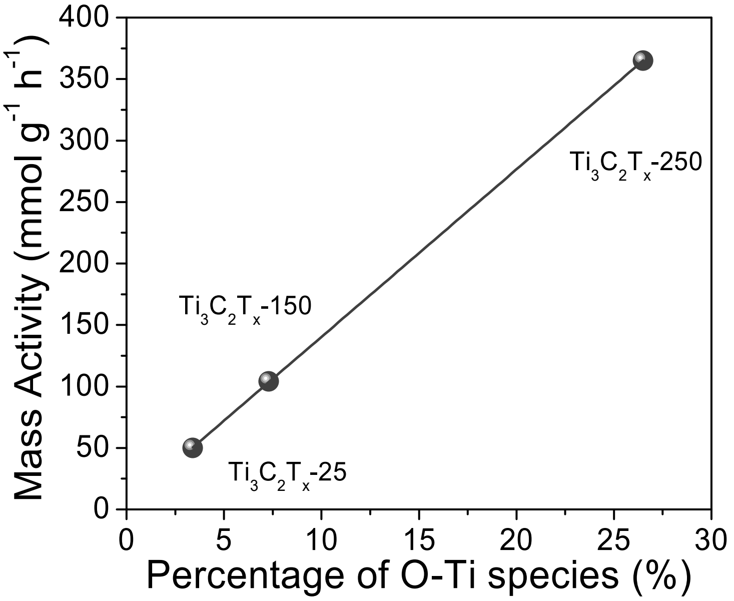

Supplementary Figure 10. The relationship between the percentage of O-Ti species and the activity of HCOOH dehydrogenation.

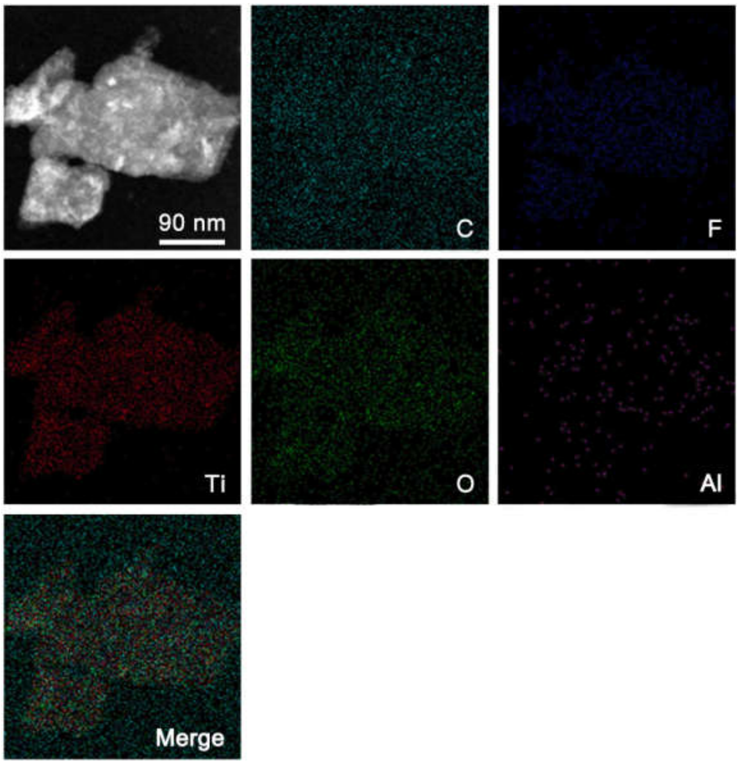

Supplementary Figure 11. HAADF-STEM and STEM-EDX elemental mapping of $Ti_3C_2T_x$-250 after catalytic test.

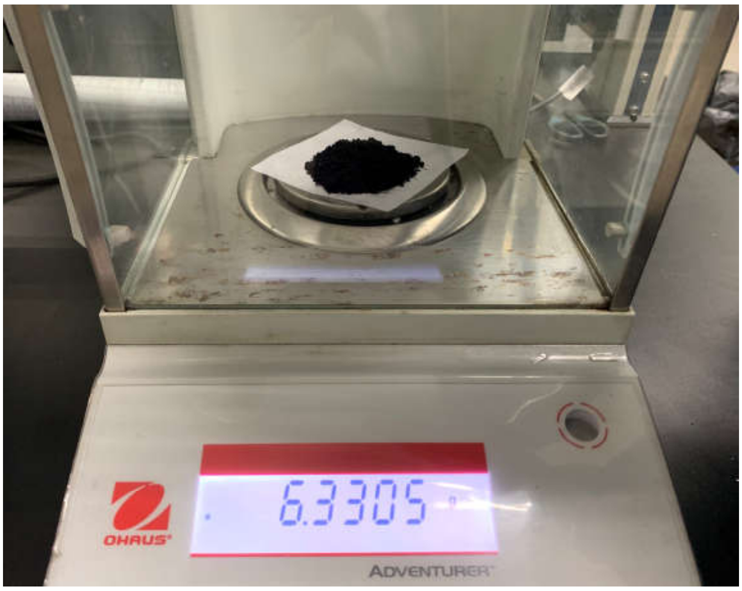

Supplementary Figure 12. The amount of $Ti_3C_2T_x$-25 prepared in one pot.

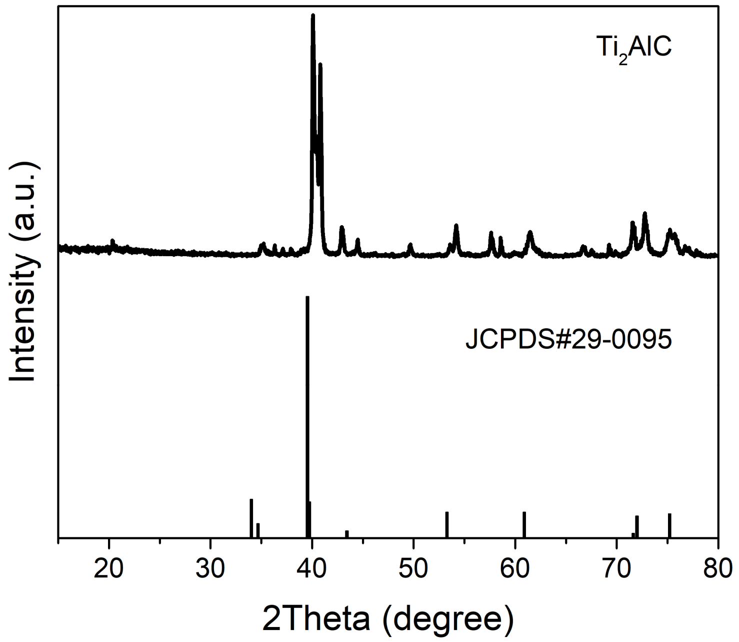

Supplementary Figure 13. XRD pattern of commercial Ti_2AlC powder.

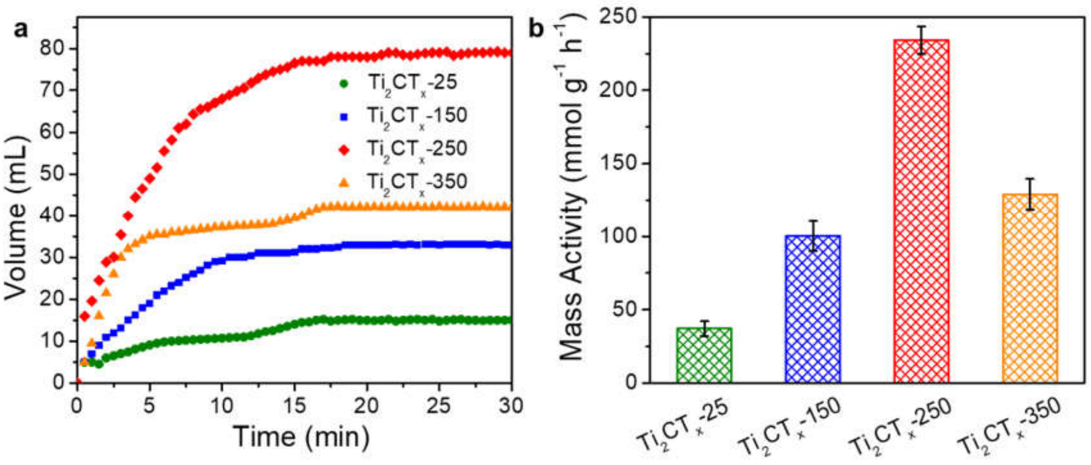

Supplementary Figure 14. (**a**) Plots of the volume of gas *vs* time at 80 °C, and (**b**) the mass activities in HCOOH dehydrogenation over Ti_2CT_x-25, Ti_2CT_x-150, Ti_2CT_x-250, and Ti_2CT_x-350.

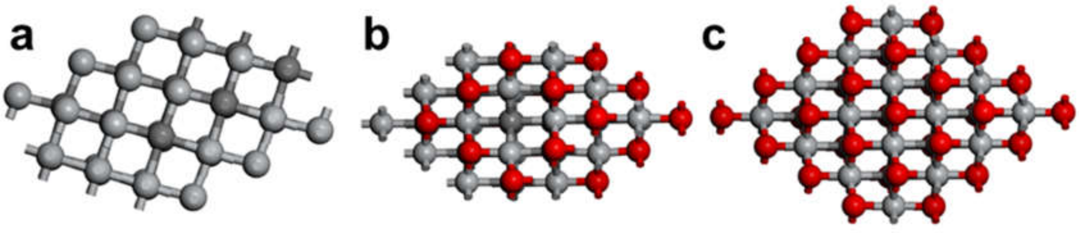

Supplementary Figure 15. The models of (**a**) Ti_3C_2 without surface oxygen atoms, (**b**) $Ti_3C_2O_2$-V with oxygen vacancies on the surface, and (**c**) $Ti_3C_2O_2$ with saturated oxygen coverage. The red, white, light gray and dark gray balls represent O, H, Ti, and C atoms, respectively.

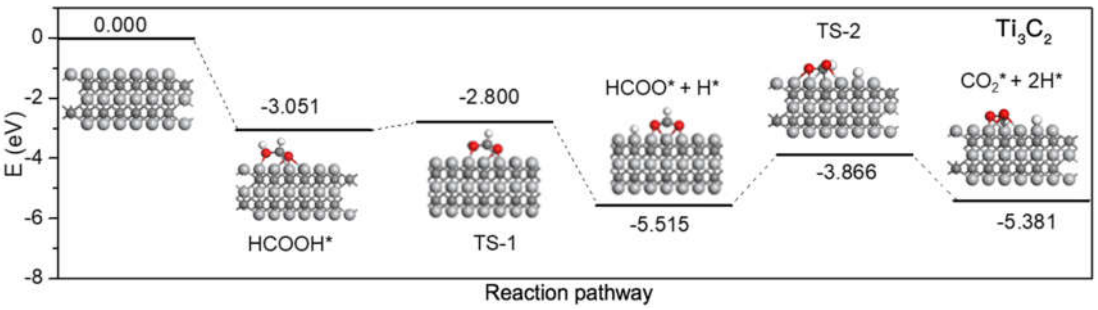

Supplementary Figure 16. Reaction paths for the dehydrogenation of HCOOH over Ti_3C_2. The red, white, light gray and dark gray balls represent O, H, Ti, and C atoms, respectively.

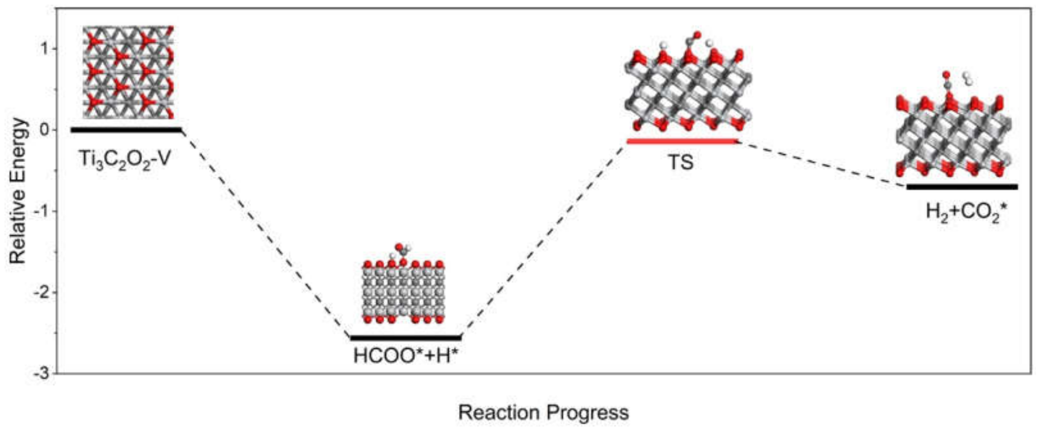

Supplementary Figure 17. Reaction paths for the dehydrogenation of HCOOH over $Ti_3C_2O_2$-V. The red, white, light gray and dark gray balls represent O, H, Ti, and C atoms, respectively.

Supplementary Table 7. The adsorption energy of CO_2 and H_2 on the surface of Ti_3C_2 and $Ti_3C_2O_2$.

Models	Ti_3C_2	$Ti_3C_2O_2$
Adsorption energy of H_2 (eV)	-0.51	-0.06
Adsorption energy of CO_2 (eV)	-2.93	-0.16

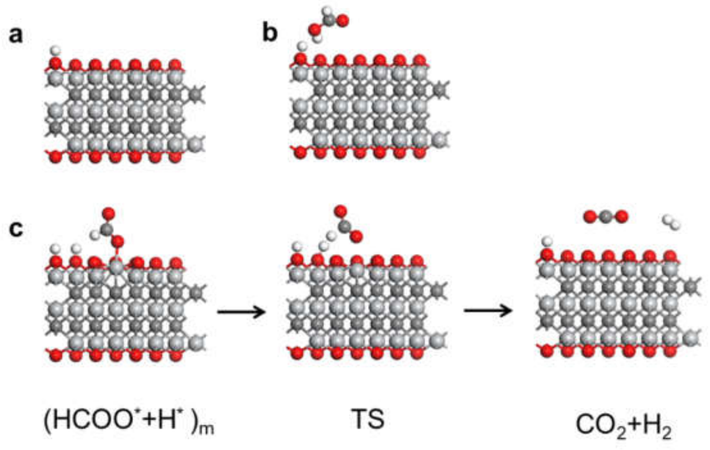

Supplementary Figure 18. (**a**) The model of $Ti_3C_2O_2$-H_1 and (**b**) the adsorption configuration of HCOOH over $Ti_3C_2O_2$-H_1. (**c**) The dehydrogenation of the adsorbed HCOOH over $Ti_3C_2O_2$-H. * and m represent as the adsorbed species and meta-stable state, respectively. $Ti_3C_2O_2$-H_1 exhibited a stronger adsorption for HCOOH with an adsorption energy of -0.53 eV than that of $Ti_3C_2O_2$ with the adsorption energy of -0.35 eV. In addition, we also simulated the process of the H_2 formation on $Ti_3C_2O_2$-H_1, where the activation energy was computed to be 0.57 eV, very close to that over $Ti_3C_2O_2$ with the value of 0.61 eV. Thus, the surface OH groups on $Ti_3C_2O_2$ might slightly boost the dehydrogenation of HCOOH. The red, white, light gray, and dark gray balls represent O, H, Ti, and C atoms, respectively.

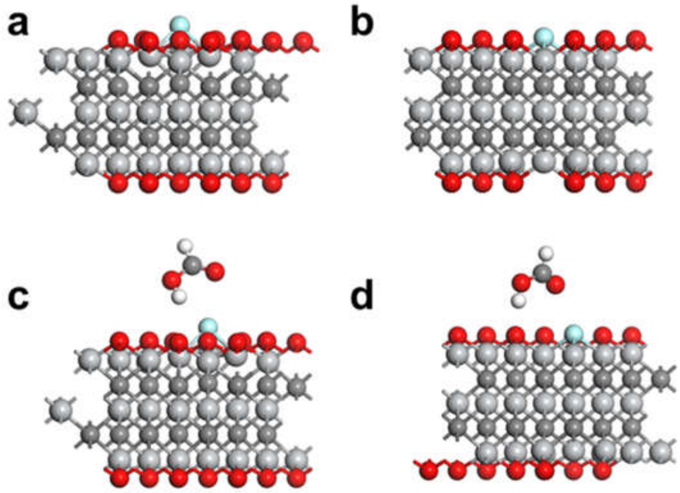

Supplementary Figure 19. The models of (**a**) $F_1@Ti_3C_2O_2$ and (**b**) $F_1@Ti_3C_2O_2$-V. The adsorption configuration of HCOOH over (**c**) $F_1@Ti_3C_2O_2$ and (**d**) $F_1@Ti_3C_2O_2$-V. $F_1@Ti_3C_2O_2$ and $F_1@Ti_3C_2O_2$-V represent a single F atom adsorbed on $Ti_3C_2O_2$ and $Ti_3C_2O_2$-V, respectively. $F_1@Ti_3C_2O_2$ exhibited a weak adsorption for HCOOH with the adsorption energy of -0.17 eV. As for $F_1@Ti_3C_2O_2$-V, a similar adsorption energy of -0.36 eV was observed compared with that for $Ti_3C_2O_2$-V with the value of -0.35 eV. Therefore, the influence of F species on HCOOH dehydrogenation was negligible in this case. The red, white, light gray, dark gray, and green balls represent O, H, Ti, C, and F atoms, respectively.

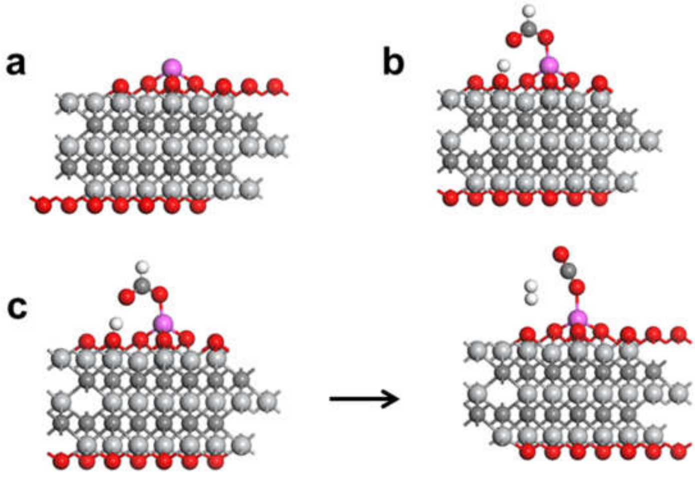

Supplementary Figure 20. (**a**) The model of $Al_1@Ti_3C_2O_2$ and (**b**) the adsorption configuration of HCOOH over $Al_1@Ti_3C_2O_2$. (**c**) The dehydrogenation of the adsorbed HCOOH over $Al_1@Ti_3C_2O_2$. HCOOH had a dissociation adsorption on the surface of $Al_1@Ti_3C_2O_2$ to generate HCOO-Al* and H* with the adsorption energy of -3.29 eV (Panel B). The subsequent dissociation of HCOO-Al* to COO-Al* was severely impeded thermodynamically with the reaction energy up to 2.46 eV (Panel C). The red, white, light gray, dark gray, and pink balls represent O, H, Ti, C, and Al atoms, respectively.

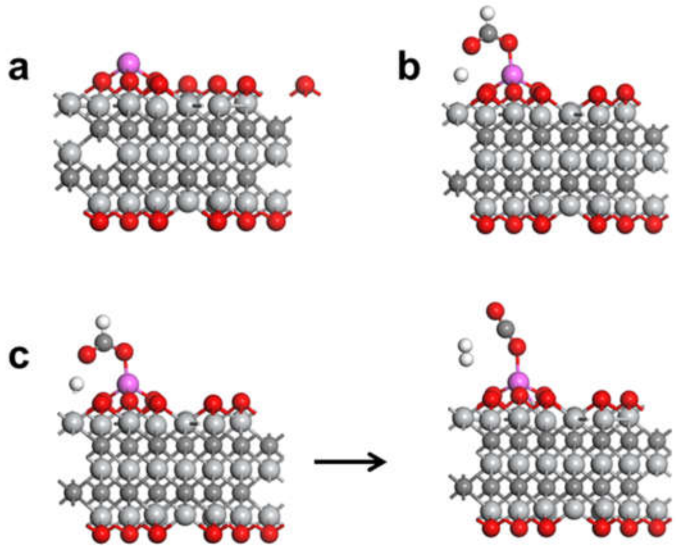

Supplementary Figure 21. (**a**) The model of $Al_1@Ti_3C_2O_2$-V and (**b**) the adsorption configuration of HCOOH over $Al_1@Ti_3C_2O_2$-V. (**c**) The dehydrogenation of the adsorbed HCOOH over $Al_1@Ti_3C_2O_2$-V. HCOOH had a dissociation adsorption on the surface of $Al_1@Ti_3C_2O_2$-V to generate HCOO-Al* and H* with the adsorption energy of -3.26 eV (Panel B). The subsequent dissociation of HCOO-Al* to COO-Al* was severely impeded thermodynamically with the reaction energy up to 2.54 eV (Panel C). The red, white, light gray, dark gray, and pink balls represent O, H, Ti, C, and Al atoms, respectively.

Supplementary References

S1. Wang, J. L. *et al.* Non-precious molybdenum-based catalyst derived from biomass: CO-free hydrogen production from formic acid at low temperature. *Energy Convers. Manage.* **164**, 122-131 (2018).

S2. Tang, C. H. *et al.* A stable nanocobalt catalyst with highly dispersed CoNx active sites for the selective dehydrogenation of formic acid. *Angew. Chem., Int. Ed.* **56**, 16616-16620 (2017).

S3. Bide, Y., Nabid, M. R. & Etemadi, B. Facile synthesis and catalytic application of selenium doped graphene/$CoFe_2O_4$ for highly efficient and noble metal free dehydrogenation of formic acid. *Int. J. Hydrogen Energy* **41**, 20147-20155 (2016).

S4. Bertini, F., Mellone, I., Ienco, A., Peruzzini, M. & Gonsalvi, L. Iron(II) complexes of the linear rac-tetraphos-1 ligand as efficient homogeneous catalysts for sodium bicarbonate hydrogenation and formic acid dehydrogenation. *ACS Catal.* **5**, 1254-1265 (2015).

S5. Kuehnel, M. F., Wakerley, D. W., Orchard, K. L. & Reisner, E. Photocatalytic formic acid conversion on CdS nanocrystals with controllable selectivity for H_2 or CO. *Angew. Chem., Int. Ed.* **54**, 9627-9631 (2015).

S6. Enthaler, S. *et al.* Exploring the reactivity of nickel pincer complexes in the decomposition of formic acid to CO_2/H_2 and the hydrogenation of $NaHCO_3$ to HCOONa. *ChemCatChem* **7**, 65-69 (2015).

S7. Bielinski, E. A. *et al.* Lewis acid-assisted formic acid dehydrogenation using a pincer-supported iron catalyst. *J. Am. Chem. Soc.* **136**, 10234-10237 (2014).

S8 Myers, T. W. & Berben, L. A. Aluminium-ligand cooperation promotes selective dehydrogenation of formic acid to H_2 and CO_2. *Chem. Sci.* **5**, 2771-2777 (2014).

S9. Koroteev, V. O., Bulushev, D. A., Chuvilin, A. L., Okotrub, A. V. & Bulusheva, L. G. Nanometer-sized MoS_2 clusters on graphene flakes for catalytic formic acid decomposition. *ACS Catal.* **4**, 3950-3956 (2014).

S10. Zell, T., Butschke, B., Ben-David, Y. & Milstein, D. Efficient hydrogen liberation from formic acid catalyzed by a well-defined iron pincer complex under mild conditions. *Chem. Eur. J.*

19, 8068-8072 (2013).

S11. Boddien, A. *et al.* Efficient dehydrogenation of formic acid using an iron catalyst. *Science* **333**, 1733-1736 (2011).

S12. Boddien, A. *et al.* ortho-Metalation of iron(0) tribenzylphosphine complexes: homogeneous catalysts for the generation of hydrogen from formic acid. *Angew. Chem., Int. Ed.* **49**, 8993-8996 (2010).

S13. Boddien, A. *et al.* Catalytic generation of hydrogen from formic acid and its derivatives: useful hydrogen storage materials. *J. Am. Chem. Soc.* **132**, 8924–8934 (2010).
